# The Burden of Provider-Initiated Preterm Birth and Associated Factors: Evidence from the Brazilian Multicenter Study on Preterm Birth (EMIP)

**DOI:** 10.1371/journal.pone.0148244

**Published:** 2016-02-05

**Authors:** Renato T. Souza, Jose G. Cecatti, Renato Passini, Ricardo P. Tedesco, Giuliane J. Lajos, Marcelo L. Nomura, Patricia M. Rehder, Tabata Z. Dias, Samira M. Haddad, Rodolfo C. Pacagnella, Maria L. Costa, Brazilian Multicenter Study on Preterm Birth study group

**Affiliations:** 1 Department of Obstetrics and Gynecology, University of Campinas (UNICAMP) School of Medicine, Campinas, SP, Brazil; 2 Department of Obstetrics and Gynecology, Jundiaí Medical School, Jundiaí, SP, Brazil; John Hunter Hospital, AUSTRALIA

## Abstract

**Background:**

About 15 million children are born under 37 weeks of gestation worldwide. Prematurity is the leading cause of neonatal deaths and short/long term morbidities, entailing consequences not only for the individual, but also their family, health agencies, facilities and all community. The provider-initiated preterm birth is currently one of the most important obstetric conditions related to preterm births, particularly in middle and high income countries, thus decreasing the need for therapeutic preterm birth is essential to reduce global prematurity. Therefore detailed knowledge on the factors associated with provider-initiated preterm birth is essential for the efforts to reduce preterm birth rates and its consequences. In this current analysis we aimed to assess the proportion of provider-initiated (pi-PTB) among preterm births in Brazil and identify associated factors.

**Methods and Findings:**

This is an analysis of a multicenter cross-sectional study with a nested case-control component called Brazilian Multicenter Study on Preterm Birth (EMIP). EMIP was conducted in 20 referral obstetric hospitals located in the three most populated of the five Brazilian regions. We analysed data of women with pi-PTB, defined as childbirth occurring at less than 37 weeks, medically indicated for maternal/fetal compromise or both; and women with term birth, childbirth at or after 37 weeks. Maternal, sociodemographic, obstetric, prenatal care, delivery, and postnatal characteristics were assessed as possible factors associated with pi-PTB, compared to term births. The overall prevalence of preterm births was 12.3%. Of these, approximately one-third of cases were initiated by the provider. Hypertensive disorders, placental abruption, and diabetes were the main maternal conditions leading to pi-PTB. Caesarean section was the most common mode of delivery. Chronic hypertension (OR 7.47; 95%CI 4.02–13.88), preeclampsia/eclampsia/HELLP syndrome (OR 15.35; 6.57–35.88), multiple pregnancy (OR 12.49; 4.86–32.05), and chronic diabetes (OR 5.24; 2.68–10.25) were the most significant factors independently associated with pi-PTB.

**Conclusions:**

pi-PTB is responsible for about one-third of all preterm births, requiring special attention. The decision-making process relative to the choice of provider-initiated birth is complex, and many factors should be elucidated to improve strategies for its prevention, including evidence-based guidelines on proper management of the corresponding clinical conditions.

## Introduction

The World Health Organization (WHO) estimates that the worldwide incidence of preterm births is 15 million annually, accounting for 11.1% of all births [1]. More recently, the prevalence of preterm births in Brazil has been estimated to range from 9.9% [1] to 12.3% [2]. The main conditions leading to preterm birth are spontaneous preterm labor, preterm premature rupture of membranes (pPROM) or provider-initiated (medically indicated) delivery, due to maternal or fetal indications [3–5]. Contributing factor vary widely among different regions or countries [6,7]. There is some indication that the substantial rise in preterm births observed during the last few decades may be explained by growing pi-PTB rates [3].

Multiple risk factors play a role in such distinct rates. Pi-PTB is recurrently associated with either maternal pathological conditions (severe preeclampsia, eclampsia, placental abruption, placenta previa, and other severe maternal clinical conditions); or fetal conditions (fetal growth restriction, fetal distress or even fetal malformations). In addition, some maternal characteristics and morbidities including age, race/ethnicity, body mass index, assisted reproductive procedures, hypertension, diabetes, and others are closely linked to a higher pi-PTB risk [4,7–10].

The WHO Multicountry Survey analysed more than 300,000 births, concluding that maternal age >35, nulliparity, previous C-section, anaemia, malaria/dengue, chronic hypertension, and preeclampsia/eclampsia were maternal conditions associated with pi-PTB. Furthermore, stillbirth and early neonatal death rates were higher in pi-PTB than in spontaneous preterm births (sPTB) [6].

Preterm birth is the current leading cause of neonatal mortality. It is also responsible for more than half of long-term neonatal morbidities [11–13]. However, death rates resulting from prematurity have not shown the same reduction as global neonatal mortality rates in recent decades [11]. A study from 192 countries showed that the lower the neonatal mortality rate, the higher the importance of prematurity in the etiology of neonatal death. Despite the decrease of neonatal mortality, the preterm component remains static. In some cases, it is even higher and most likely attributed to pi-PTB [14].

A decrease in pi-PTB is essential to reduce global prematurity and its resultant neonatal morbidity and mortality [4,13], particularly in middle-, high- and very high-income countries [6], as part of effective preventive strategies for the preconception or postconceptional periods [15–17]. It is then fundamental to investigate the underlying conditions related to pi-PTB in different backgrounds [15,18]. Therefore, we intend to identify the prevalence, characteristics and main factors associated with pi-PTB, as well as corresponding perinatal outcomes in the Brazilian Multicentre Study of Preterm Birth (EMIP) [19,20].

## Methods

### Ethics statement

The proposal for this study has been reviewed and approved by the National Council for Ethics in Research (CONEP) and by the Institutional Review Board of each site. Before enrolment, an individual Informed Consent form was signed by each subject after understanding and accepting the study conditions. The confidentiality of identity was ensured regardless of whether the women participated in the study or not. The study totally complies with The Declaration of Helsinki, following Brazilian National Health Council Resolution 196/96.

The Review Boards of the following institutions reviewed and approved this study: Maternidade Climério de Oliveira (Salvador, BA), Maternidade Escola Assis Chateaubriand (Fortaleza, CE), Hospital Universitário da Universidade Federal do Maranhão (Sao Luis, MA), Instituto de Saúde Elídio de Almeida (Campina Grande, PB), Hospital Universitário Lauro Wanderley da Universidade Federal da Paraíba (Joao Pessoa, PB), Instituto de Medicina Integral Prof. Fernando Figueira (Recife, PE), Hospital das Clınicas da Universidade Federal de Pernambuco (Recife, PE), Hospital das Clınicas da Universidade Federal do Paraná (Curitiba, PR), Instituto Fernandes Figueira (Rio de Janeiro, RJ), Hospital das Clinicas da Universidade Federal do Rio Grande do Sul (Porto Alegre, RS), Faculdade de Medicina de Botucatu da Universidade Estadual Paulista (Botucatu, SP), Hospital da Mulher da Universidade Estadual de Campinas (Campinas, SP), Maternidade Escola de Vila Nova Cachoeirinha (São Paulo, SP), Hospital Estadual de Sumaré (Sumaré, SP), Faculdade de Medicina de Jundiaí (Jundiaí, SP), Hospital das Clınicas da Faculdade de Medicina de Ribeirão Preto da Universidade de São Paulo (Ribeirão Preto, SP), Santa Casa de Limeira (Limeira, SP), Santa Casa de São Carlos (São Carlos, SP), Casa Maternal Leonor Mendes de Barros (São Paulo, SP), and Hospital São Paulo da Universidade Federal de São Paulo (São Paulo, SP).

### Study design, population and sample size

The Brazilian Multicentre Study on Preterm Birth (EMIP) took place in 20 referral obstetric hospitals located in the three most populated regions of Brazil. Methodological details of this study were published elsewhere [19,21]. Briefly, this cross-sectional study carried out surveillance of 33,740 deliveries from April 2011 to July 2012. A dataset of all preterm deliveries and preterm infants was established to allow the descriptive evaluation of each one of the three components (spontaneous preterm birth, preterm birth following a premature rupture of membranes, and a therapeutic or provider-initiated preterm birth). In addition, we also implemented a nested unmatched case-control component to compare preterm births with a term-birth sample, considering the limitation for a cohort study and getting information for all births during the period. A term birth immediately after a preterm birth was included as a proxy for a randomly selected control group, if the women agreed to participate, until reaching the previously estimated sample size. Otherwise, the next term birth was approached. Sample calculation for the whole study was based on the Brazilian preterm birth prevalence of 6.5% in 2006 and at least 1054 subjects per group for the case-control component [2,19,22].

During the data collection period, each participating centre monitored preterm births continuously to identify women eligible for the study. Once identified, women were invited to participate. After receiving explanations, agreeing to participate and signing a consent term, these women were enrolled. Information was collected through three sources: postpartum interview using a structured questionnaire and medical chart review. Neonatal data was collected up to hospital discharge or up to sixty days after birth if the neonate remained admitted.

### Variables and outcomes

The main outcome for this analysis was pi-PTB, defined as childbirth occurring at less than 37 weeks, medically indicated for maternal/fetal compromise or both; or term birth, childbirth at or after 37 weeks. Preterm birth were classified as extremely preterm (before 28 completed weeks), very preterm (from 28 to 31 completed weeks) and moderately preterm (from 32 to 36 completed weeks), according to the World Health Organization [1]. Data on another separate category including only late preterm births (from 34 to 36 completed weeks) was reported [23]. Other secondary outcomes were admission to neonatal intensive care unit (NICU); Apgar score < 7 at 5 minutes; low birth weight (<2500g); and neonatal mortality.

Variables assessed as potential predictors of pi-PTB were related to sociodemographic characteristics, working status, maternal weight assessment, reproductive and obstetric history, prenatal care (including adequate number of prenatal care visits for gestational age, previously defined by the Brazilian Ministry of Health [24]), lifestyle and habits, data on short cervix (clinical history or cervical length < 25 mm measured between 14 to 24 weeks by vaginal ultrasound scan), suspected cervical insufficiency (clinical or ultrasound signs), cerclage during pregnancy, uterine fibroid, vaginal bleeding, oligohydramnios or polyhydramnios, fetal malformation, fetal growth restriction, multiple pregnancy, features of maternal and fetal care, and maternal and/or fetal indication for delivery. For defining all these variables we used a pragmatic approach using information reported in medical records by providers of care considering clinical and ultrasonographic aspects according to the standard procedures of each participating centre.

### Data quality

Several procedures were adopted to ensure high-quality data and reliable information, including preparatory meetings, use of a detailed manual of operation, site visits, technical visits to participating centers, closely monitored data collection and data entry, concurrent query management, inconsistency checks, and database correction [20].

### Statistical analysis

Initially the pi-PTB prevalence was estimated among all preterm births occurring in participating centres during the data collection period. Estimation was stratified based on geographical region, gestational age, delivery indications, and clinical management. Delivery indications, maternal and neonatal outcomes were distributed according to gestational age, and evaluated by χ^2^ tests.

Bivariate analyses were then conducted to identify and estimate risks of pi-PTB, using the Odds Ratio (OR) with 95% confidence intervals (CI) for each predictor (sociodemographic, obstetric history and current pregnancy conditions), adjusting for clustering effect design. To identify factors independently associated with pi-PTB and to control for confounding, a multivariate analysis using non-conditional multiple logistic regression was performed, reporting the estimated adjusted OR_adj_ with a 95% CI. Backward stepwise regression was performed including all variables in the logistic regression model. Missing data were excluded from analysis and are detailed reported in the tables´ footnotes. Each centre was considered a cluster. Low intracluster correlation coefficients for variables were considered appropriate, since centers heterogeneity strengthen sample representation [21].

Statistical analysis was carried out using SPSS version 20.0 (SPSS, Chicago, IL, USA) and Stata version 7.0 (StataCorp, College Station, TX, USA). A *p*-value <0.05 was considered significant.

## Results

**Fig 1:**
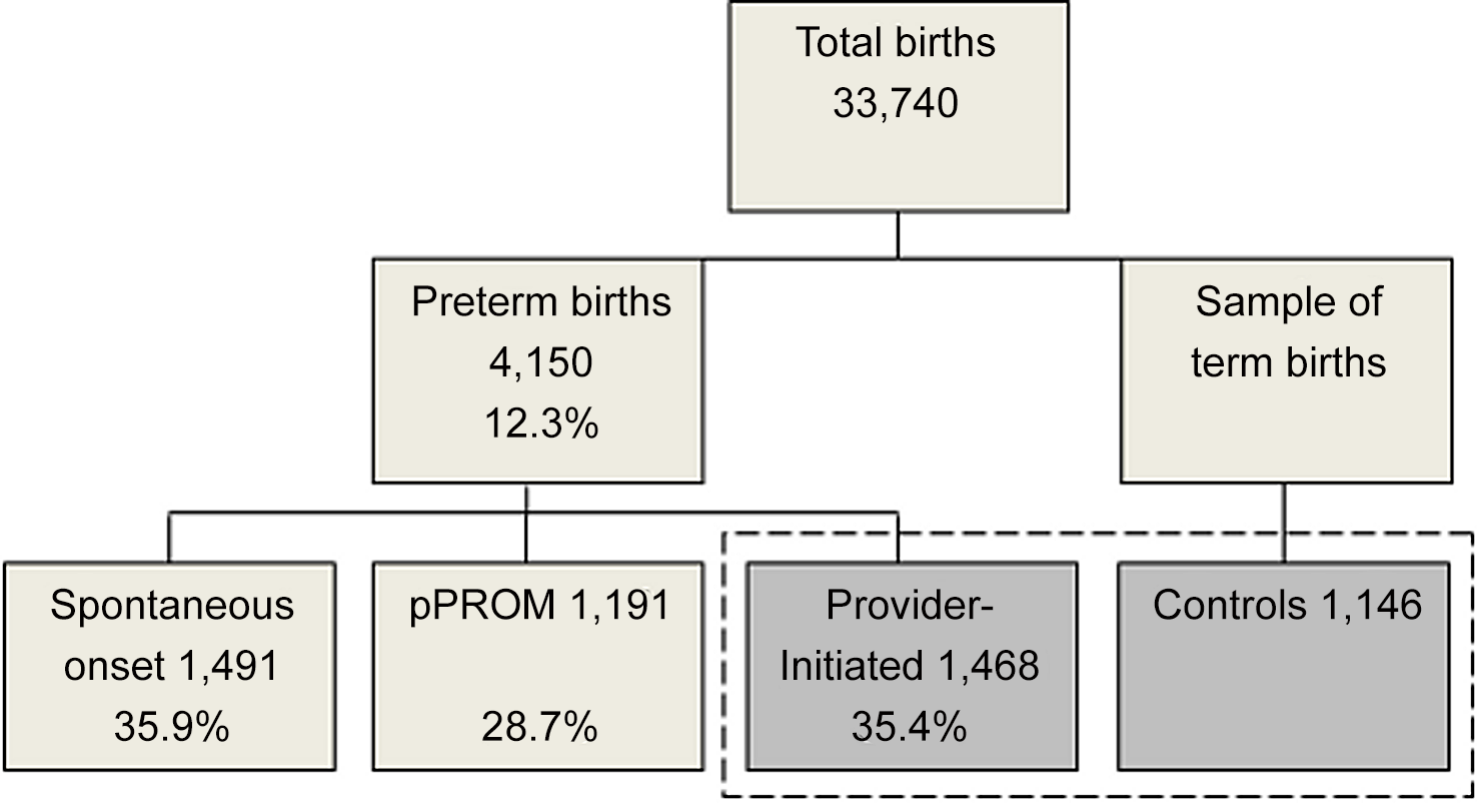
Flow chart of subjects in the Brazilian Multicentre Study on Preterm Birth.

EMIP carried out surveillance of 33,740 births in 20 participating centres, identifying 1,468 pi-PTBs, and selecting a sample of 1,146 term births (Fig 1). The total prevalence of preterm birth was 12.3%. During the study period, 9.37% of women having preterm births in the participating centres were not enrolled due to some very few refusals and mainly due to hospital discharge before they could be approached by the interviewers.

The proportion of pi-PTB among all preterm births was 35.4%, ranging from 34.7% in the Southeast to 39.6% in the South of Brazil. The Southeast region accounted for the majority of the pi-PTB (54.1%), while only 14% occurred in the South in this sample (Table 1). Multiple pregnancies occurred in 7.7% of pi-PTB, representing 25.9% of all preterm multiple births identified. More than three-quarters (76.7%) of births occurred between 32 and 36 weeks, while the lowest proportion occurred in pregnancies less than 28 weeks (6.9%). The late pi-PTB (34–36 weeks) corresponded to almost 60% of them.

**Table 1:** Prevalence of provider-initiated preterm births in a sample of selected tertiary referral Brazilian maternities according to geographical region, gestational age and delivery indications.

	Provider-initiated Preterm births n (%)	Proportion of provider-initiated delivery among all preterm deliveries (%)
**BRAZIL (total)**	**1,468 (100)**	**35.4**
**Region**		
**Southeast**	794 (54.1)	34.7
**Northeast**	468 (31.9)	34.9
**South**	206 (14.0)	39.6
**Gestational age at delivery**		
**<28 weeks**	101 (6.9)	32.8
**28–31 weeks**	241 (16.4)	42.1
**32–36 weeks**	1126 (76.7)	34.4
***34–36 weeks***	*859 (58*.*5)*	*33*.*0*
**Multiple pregnancy**	113 (7.7)	25.9
**Indication of pregnancy interruption** (a)		
**Maternal**	670 (45.9)	
**Fetal**	370 (25.4)	
**Maternal and Fetal**	419 (28.7)	
**Maternal indication for interruption** * [n = 1084] (b)		
**Preeclampsia**	631 (58.2)	
**Chronic hypertension**	166 (15.3)	
**Gestational hypertension**	140 (12.9)	
**HELLP syndrome**	102 (9.4)	
**Placental abruption**	83 (7.7)	
**Diabetes**	79 (7.3)	
**Placenta previa**	36 (3.3)	
**Non-obstetric infection**	16 (1.5)	
**Maternal cardiac disease**	12 (1.1)	
**Chorioamnionitis**	6 (0.6)	
Others**	96 (8.8)	

*Indications are not mutually exclusive.

**Miscalculation of gestational age, maternal injury, pulmonary hypertension, autoimmune disease, delivery by maternal request.

Missing information for: (a) 9; (b) 5 cases.

Exclusive maternal condition was the main indication for delivery in 45.9% of pi-PTB. Hypertensive disorders (preeclampsia– 58.2%, chronic hypertension– 15.3%, gestational hypertension– 12.9%, and HELPP syndrome– 9.4%) were the most common indications, regardless of gestational age (Tables 1 and 2). HELLP syndrome, placental abruption and chorioamnionitis occurred more frequently before 32 weeks. Fetal distress and fetal growth restriction (FGR) were the most important fetal indications in all gestational ages (Table 2). It shows that major fetal surveillance methods used were Doppler ultrasound and cardiotocography, while fetal movement counting was rarely employed. Table 2 also shows that only 58.2% of women with pregnancies interrupted at less than 28 weeks received antenatal corticosteroids (ACS). However, almost three-quarters of women between 28–31 weeks received corticosteroids for fetal lung maturation at least once during pregnancy.

**Table 2:** Fetal and maternal indications for pregnancy interruption and characteristics of obstetric care according to gestational age in provider-initiated preterm births (n = 1,468).

	Gestational age				
Indications for pregnancy interruption and characteristics of obstetric care	<28 weeks	28–31 weeks	32–36 weeks	p-value	34–36 weeks
**Maternal indication for interruption** [n = 1084] (a)					
**Preeclampsia**	39 (54.9)	120 (65.9)	472 (56.8)	0.157	358 (55.8)
**Chronic hypertension**	8 (11.3)	32 (17.6)	126 (15.2)	0.452	98 (15.3)
**HELLP syndrome**	10 (14.1)	31 (17.0)	61 (7.3)	**<0.001**	38 (5.9)
**Placental abruption**	12 (16.9)	17 (9.3)	54 (6.5)	**0.010**	35 (5.5)
**Gestational hypertension**	6 (8.5)	22 (12.1)	112 (13.5)	0.338	88 (13.7)
**Placenta previa**	4 (5.6)	8 (4.4)	24 (2.9)	0.433	21 (3.3)
**Eclampsia**	2 (2.8)	4 (2.2)	29 (3.5)	0.571	25 (3.9)
**Diabetes**	0 (-)	5 (2.7)	74 (8.9)	0.086	64 (10.0)
**Chorioamnionitis**	2 (2.8)	1 (0.5)	3 (0.4)	**0.045**	3 (0.5)
**Non-obstetrical infection**	0 (-)	2 (1.1)	14 (1.7)	0.537	13 (2.0)
**Maternal cardiopathy**	0 (-)	3 (1.6)	9 (1.1)	0.503	9 (1.4)
**Placental insufficiency**	1 (1.4)	3 (1.6)	8 (1.0)	0.713	3 (0.5)
**Others**^#^	5 (7.0)	2 (1.1)	77 (9.3)	**<0.001**	66 (10.3)
**Fetal indication for interruption** [n = 1427] (b)					
**Fetal distress**	33 (47.8)	89 (60.5)	326 (57.2)	0.380	221 (54.4)
**Fetal growth restriction**	15 (21.7)	61 (41.5)	189 (33.2)	0.051	129 (31.8)
**Fetal malformation**	11 (15.9)	6 (4.1)	52 (9.1)	**0.026**	38 (9.4)
**Others**	25 (36.2)	41 (27.9)	131 (23.0)	**0.047**	100 (24.6)
**Fetal monitoring methods** [n = 1348] (c)					
**Doppler ultrasound**	57 (60.6)	155 (68.3)	612 (59.6)	0.253	439 (56.8)
**Cardiotocography**	30 (31.9)	118 (52.0)	674 (65.6)	**<0.001**	519 (67.1)
**Fetal biophysical profile**	28 (29.8)	88 (38.8)	318 (31.0)	0.088	227 (29.4)
**Fetal movement counting**	1 (1.1)	10 (4.4)	47 (4.6)	0.319	34 (4.4)
**Antenatal corticosteroids (%)** [n = 1400] (d)	57 (58.2)	164 (74.5)	375 (34.7)	**<0.001**	203 (24.5)
**Multiple courses of antenatal corticosteroids** [n = 560] (e)	2 (3.7)	10 (6.5)	29 (8.3)	0.385	16 (8.4)
**An attempt to treat clinical condition that motivated interruption** [n = 1428] (f)	58 (58.0)	163 (69.7)	606 (55.4)	**0.006**	433 (52.0)
**Duration of treatment (mean of days)** [n = 796] (g)	5.7 (±5.9) (4.3–7.1)	6.7 (±14.3) (3.8–9.6)	6.8 (±13.7) (5.1–8.6)		7.0 (±14.2)

#Miscalculation of gestational age, maternal injury, pulmonary hypertension, autoimmune disease, delivery by maternal request.

P-values in bold mean they are statistically significant.

Missing information for: (a) 5; (b) 3; (c) 120; (d) 68; (e) 36; (f) 40; (g) 31.

**Fig 2:**
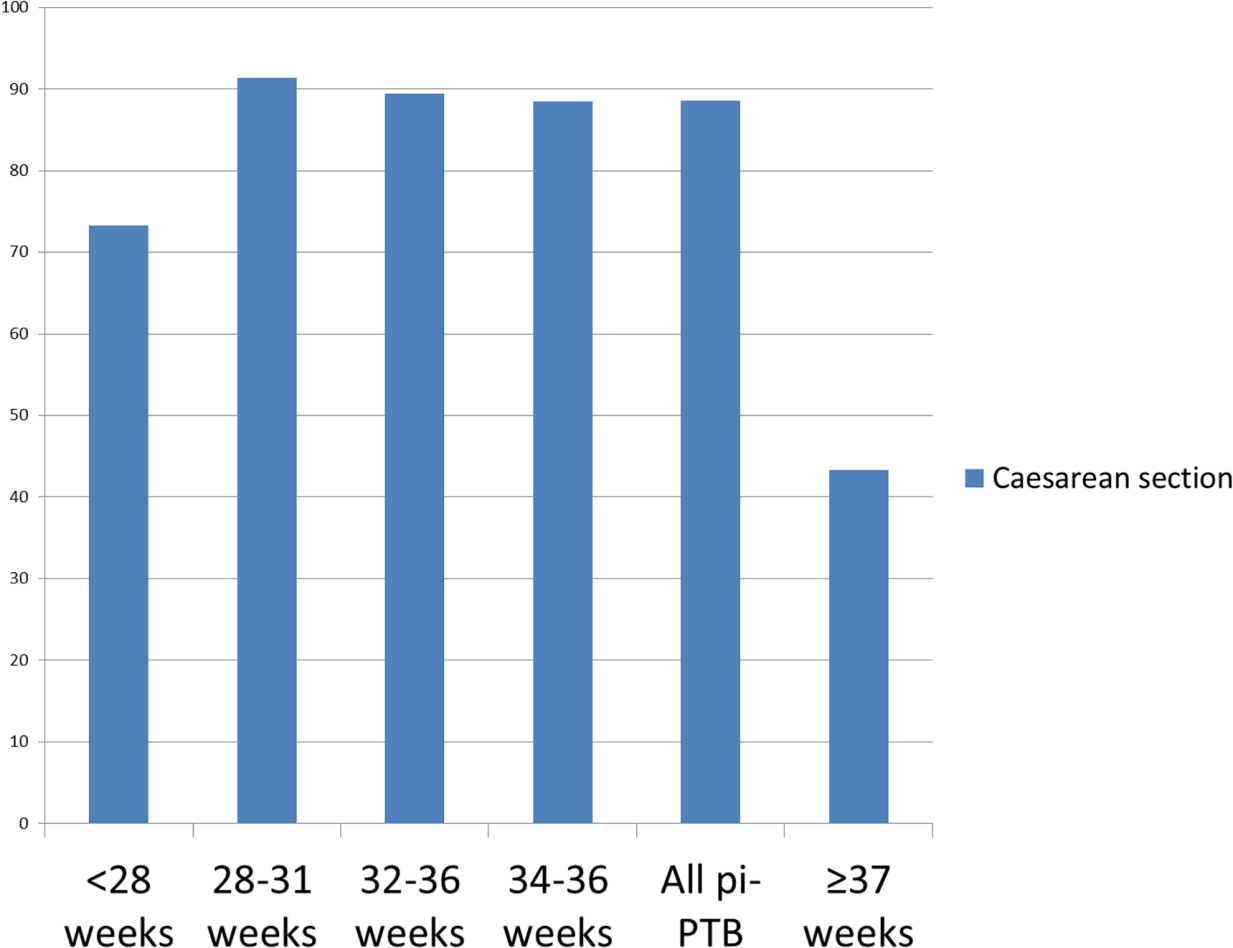
Caesarean section rates according to gestational age in provider-initiated preterm birth (n = 1,468) and term births. Mode of delivery according to the World Health Organization´s gestational age categories are significantly different (p<0.001).

**Fig 3:**
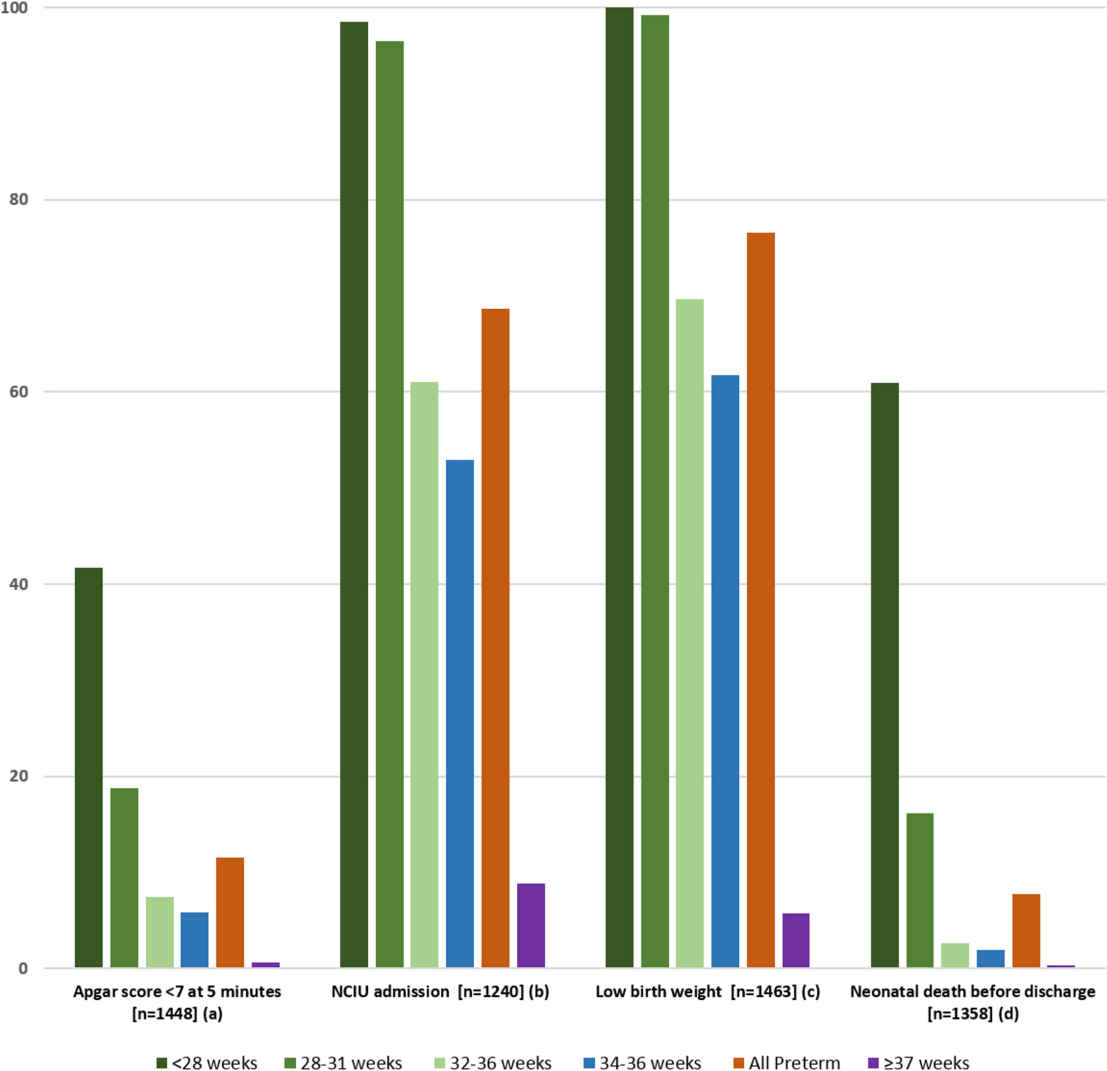
Neonatal outcomes according to gestational age in provider-initiated preterm birth (n = 1,468) and term births. Missing information for: (a) 20; (b) 131; (c) 5; (d) 13. All differences are statistically significant (p<0.001).

Fig 2 shows that the proportion of vaginal delivery was only 11.4% in pi-PTB and 56.7% in term births. The prevalence of vaginal delivery before 28 weeks doubled in comparison to other gestational ages, although it was only 26.7%. Neonatal outcomes in preterm groups were much worse than in the term group, even comparing the 34–36 week group to the term group (Fig 3). Neonatal death before discharge was more than 200 times higher in the subgroup under 28 weeks and 54 times higher in the 28–31 week subgroup than in the term group. The difference is still significant between the 34–36 week group and the term group (6.3-fold).

Regarding tests supporting the decision to deliver and obstetric care characteristics according to indication for pregnancy interruption among pi-PTB, Doppler ultrasound was the most frequently used test in fetal or maternal/fetal indications (43.8% and 46.4%, respectively). When maternal compromise indicated pregnancy interruption, haematological (27.7%) and liver function panel (23.5%) were the most common tests ordered. The attempts to treat the clinical condition causing pi-PTB and ACS use were lower in fetal (29.2% and 32.8%, respectively) than in maternal indication (66.9% and 43.3%, respectively) or maternal and fetal (68.5% and 49.6%, respectively). However, no difference was identified in treatment duration between both conditions (data not shown).

Case assessment of estimated pi-PTB risk showed that maternal age ≤ 19 had a 28% lower risk, while > 12 years of schooling had a 64% higher risk, and maternal age ≥ 35 had a 99% higher risk. Furthermore, having children aged 5 years or younger exerted a protective effect. On the other hand, some obstetric conditions were associated with increased pi-PTB risk, e.g. previous C-section, inter-pregnancy interval ≤ 12 months, previous preterm birth and previous newborn under 2500g (Tables 3 and 4).

**Table 3:** Risk estimates for provider-initiated preterm birth according to some maternal sociodemographic conditions.

Socio-demographic conditions	CASES	CONTROLS	OR* (95% CI)
	Provider-initiated N (%)	Term birth N (%)	
**Maternal age (years)** (a)			
**• ≤ 19**	183(12.5)	211 (18.4)	**0.72 0.61–0.85]**
**• 20–34**	980 (66.8)	809 (70.6)	1.00
**• ≥ 35**	304 (20.7)	126 (11.0)	**1.99 [1.48–2.68]**
**Skin colour/ethnicity**			
**• White**	675 (46.0)	451 (39.4)	1.00
**• Other**	793 (54.0)	695 (60.6)	0.76 [0.54–1.07]
**Marital status**			
**• With a partner**	1175 (80.0)	919 (80.2)	1.00
**• Without a partner**	293 (20.0)	227 (19.8)	1.01 [0.83–1.23]
**Household** (b)			
**• Urban**	1332 (91.1)	1021 89.5)	1.00
**• Rural**	130 (8.9)	120 (10.5)	0.83 [0.45–1.52]
**Schooling (years)** (c)			
**• ≤8**	546(37.8)	420(37.2)	1.05 [0.93–1.30]
**• 9–12**	740 (51.3)	629 (55.7)	1.00
**• >12**	157 (10.9)	81 (7.2)	**1.64 [1.23–2.20]**
**Paid work in pregnancy** (d)			
**• No**	843 (57.9)	690 (60.3)	1.00
**• Yes**	612 (42.1)	455 (39.7)	1.10 [0.87–1.39]
**Workload (daily)** [n = 1055] (e)			
**• ≤8 hours**	445 (73.8)	324 (71.7)	1.00
**• >8 hours**	158 (26.5)	128 (28.3)	0.90 [0.68–1.20]
**Children under 5 years** (f)			
**• No**	1136 (77.5)	821 (71.7)	1.00
**• Yes**	330 (22.5)	324 (28.3)	**0.74 [0.60–0.90]**
**Total**	**1,468 (100)**	**1,146 (100)**	

OR*: Odds Ratio adjusted for the cluster effect design; **CI:** confidence interval.

Values in bold mean they are statistically significant.

Missing information for: (a) 1; (b) 11; (c) 418; (d) 14; (e) 12; (f) 3.

**Table 4:** Risk estimates for provider-initiated preterm birth according to maternal obstetric history.

Obstetric history	CASES	CONTROLS	OR* (95% CI)
	Provider-initiated N (%)	Term birth N (%)	
**Parity**			
**• Nulliparous**	670 (45.6)	527 (46.0)	1.00
**• 1–2 deliveries**	589 (40.1)	491 (42.8)	0.94 [0.78–1.14]
**≥ 3 deliveries**	209 (14.2)	128 (11.2)	1.28 [0.94–1.76]
**Total**	**1,468 (100)**	**1,146 (100)**	
Previous caesarean section*			
**• No**	477 (52.4)	416 (62.4)	1.00
**• Yes**	433 (47.6)	251 (37.6)	**1.50 [1.22–1.85]**
Previous abortion*			
**• No**	540 (59.3)	435 (65.2)	1.00
**• Yes**	370 (40.7)	232 (34.8)	1.28 [1.00–1.66]
**Inter-pregnancy interval** * (a)			
**• > 12 months**	819 (91.2)	622 (94.8)	1.00
**• ≤ 12 months**	79 (8.8)	34 (5.2)	**1.76 [1.22–2.56]**
Previous cerclage* (b)			
**• No**	888 (98.8)	656 (98.9)	1.00
**• Yes**	11 (1.2)	7 (1.1)	1.16 [0.39–3.49]
Previous preterm birth* (c)			
**• No**	641 (71.0)	565 (84.8)	1.00
**• Yes**	262 (29.0)	101 (15.2)	**2.29 [1.79–2.93]**
Previous pPROM* (d)			
**• No**	824 (91.8)	599 (90.3)	1.00
**• Yes**	74 (8.2)	64 (9.7)	0.84 [0.55–1.29]
Previous newborn under 2500g* (e)			
**• No**	656 (72.7)	571 (87.2)	1.00
**• Yes**	246 (27.3)	84 (12.8)	**2.55 [2.00–3.25]**
**Total**	**910 (100)**	**667 (100)**	

OR*: Odds Ratio adjusted for the cluster design effect; **CI:** confidence interval.

*: excluded primigravida from analysis.

Values in bold mean they are statistically significant.

Missing information for: (a) 23; (b) 15; (c) 8; (d) 16; (e) 20.

Table 5 shows conditions associated with increased pi-PTB risk: fetal growth restriction (12-fold), multiple pregnancy (9-fold), preeclampsia/eclampsia/HELLP (9-fold), oligohydramnios (7-fold), fetal malformation (5-fold), chronic hypertension (5-fold), chronic diabetes (4-fold), systemic lupus erythematosus (5-fold), prenatal care not only in primary health unit (2-fold), polyhydramnios (2-fold), gestational hypertension (2-fold), gestational diabetes (2-fold), inadequate number of prenatal care visits (2-fold), obesity (1.8-fold), smoking during first and second trimester (1.8-fold), overweight (1.4-fold), vaginal bleeding (1.4-fold) and weight gain ≤7kg (1.3-fold). Women who gained more than 12kg during pregnancy and initial BMI < 18.5kg/m^2^ were at lower risk for pi-PTB.

**Table 5:** Risk estimates for provider-initiated preterm birth according to some conditions during pregnancy.

Conditions during pregnancy	CASES	CONTROLS	OR* (95% CI)
	Provider-initiated N (%)	Term birth N (%)	
**Prenatal care** [n = 2614]			
**• Yes**	1439 (98.0)	1130 (98.6)	1.00
**• No**	29 (2.0)	16 (1.4)	1.42 [0.54–3.76]
**Healthcare facility used for prenatal care** [n = 2569]			
**• Only PHU**	643 (44.7)	715 (63.3)	1.00
**• PHU + hospital**	250(17.4)	93 (8.2)	**2.99 1.92–4.67]**
**• Only hospital**	345(24.0)	234 (20.7)	**1.64 1.18–2.28]**
**• Other**	201 (14.0)	88 (7.8)	**2.54 [1.48–4.36]**
**Initiation of prenatal care** [n = 2218] (a)			
**• First trimester**	792 (64.3)	645 (65.4)	1.00
**• Second and third trimester**	440 (35.7)	341 (34.6)	1.05 [0.79–1.40]
**Adequacy of number of prenatal care visits** [n = 2323] (b)			
**• Adequate**	761 (59.5)	792 (75.8)	1.00
**• Inadequate**	517 (40.5)	253 (24.2)	**2.13 [1.57–2.88]**
**Weight gain in pregnancy** [n = 2283] (c)			
**• ≤ 7kg**	360(28.4)	221 (21.7)	**1.31 1.05–1.64]**
**• 8–12 kg**	407 (32.1)	328 (32.3)	1.00
**• > 12kg**	500 (39.5)	467 (46.0)	**0.66 [0.53–0.81]**
**Initial body mass index** [n = 2295] (d)			
**• <18,5 kg/m**^**2**^	56 (4.4)	84 (8.3)	**0.62 [0.43–0.88]**
**• 18,5–24,99 kg/m**^**2**^	614 (47.8)	571 (56.5)	1.00
**• 25–29.99 kg/m**^**2**^	346 (26.9)	218 (21.6)	**1.47 [1.20–1.81]**
**• ≥ 30 kg/m**^**2**^	269 (20.9)	137 (13.6)	**1.82 [1.44–2.31]**
**Final body mass index** [n = 2196] (e)			
**• <18,5 kg/m**^**2**^	3 (0.2)	1 (0.1)	2.83 [0.23–34.19]
**• 18,5–24,99 kg/m**^**2**^	208 (16.9)	196(20.4)	1.00
**• 25–29.99 kg/m**^**2**^	422 (34.2)	400 (41.5)	0.99 [0.77–1.29]
**• ≥ 30 kg/m**^**2**^	600 (48.7)	366 (38.0)	**1.54 [1.11–2.16]**
**Physical effort** [n = 2595] (f)			
**• No or rarely**	1184 (81.5)	896 (78.4)	1.00
**• Yes (often)**	268 (18.5)	247 (21.6)	0.82 [0.65–1.03]
**Smoking (daily)** [n = 2614]			
**• No**	1301 (88.6)	1023 (89.3)	1.00
**• ≤ 10 cigarettes**	119(8.1)	84 (7.3)	1.11 [0.80–1.55]
**• > 10 cigarettes**	48 (3.3)	39 (3.4)	0.97 [0.59–1.58]
**Smoking until (trimester)** [n = 2614]			
**• Never or not in pregnancy**	1301 (88.6)	1023 (89.3)	1.00
**• First and second**	62(4.2)	27 (2.4)	**1.81 1.03–3.16]**
**• Third**	105 (7.2)	96 (8.4)	0.88 [0.72–1.07]
**Urinary tract infection** [n = 2110] (g)			
**• No**	823 (69.0)	645 (70.3)	1.00
**• Yes**	370 (31.0)	272 (29.7)	1.07 [0.84–1.35]
**Short cervix (<25mm; US)** [n = 1163] (h)			
**• No**	679 (99.1	474 (99.2)	1.00
**• Yes**	6 (0.9)	4 (0.8)	1.05 [0.28–3.93]
**Suspected cervical insufficiency (clinical or US)** [n = 2291] (i)			
**• No**	1302 (99.5)	976 (99.4)	1.00
**• Yes**	7 (0.5)	6 (0.6)	0.87 [0.27–2.81]
**Cerclage** [n = 2348] (j)			
**• No**	1330 (99.3)	1003 (99.5)	1.00
**• Yes**	10 (0.7)	5 (0.5)	1.51 [0.45–5.10]
**Uterine fibroid** [n = 2315] (k)			
**• No**	1286 (97.5)	981 (98.5)	1.00
**• Yes**	33 (2.5)	15 (1.5)	1.68 [0.81–3.48]
** Vaginal bleeding** [n = 2610] (l)			
**• No**	1141 (77.9)	957 (83.6)	1.00
**• Yes**	324 (22.1)	188 (16.4)	**1.45 [1.18–1.77]**
**Anaemia** [n = 2571] (m)			
**• No**	1044 (72.7)	783 (69.0)	1.00
**• Yes**	393 (27.3)	351 (31.0)	0.84 [0.70–1.01]
**Chronic Hypertension** [n = 2515] (n)			
**• No**	1233 (85.9)	1049 (97.2)	1.00
**• Yes**	203 (14.1)	30 (2.8)	**5.76 [3.83–8.65]**
**Chronic Diabetes** [n = 2612] (o)			
**• No**	1399 (95.4)	1132 (98.9)	1.00
**• Yes**	68 (4.6)	13 (1.1)	**4.23 [1.78–10.08]**
**Gestational diabetes** [n = 2515] (n)			
**• No**	1300 (90.5)	1030 (95.5)	1.00
**• Yes**	136 (9.5)	49 (4.5)	**2.20 [1.16–4.16]**
**Gestational hypertension** [n = 2515] (n)			
**• No**	1236 (86.1)	1009 (93.5)	1.00
**• Yes**	200 (13.9)	70 (6.5)	**2.33 [1.10–4.94]**
**Preeclampsia/Eclampsia/HELLP** [n = 2515] (n)			
**• No**	793 (55.2)	989 (91.7)	1.00
**• Yes**	643 (44.8)	90 (8.3)	**8.91 [3.96–20.03]**
**Hypo/Hyperthyroidism** [n = 2515] (n)			
**• No**	1402 (97.6)	1062 (98.4)	1.00
**• Yes**	34 (2.4)	17 (1.6)	1.51 [0.71–3.23]
**HIV** [n = 2515] (n)			
**• No**	1424 (99.2)	1061 (98.3)	1.00
**• Yes**	12 (0.8)	18 (1.7)	0.50 [0.24–1.04]
**Cardiac disease** [n = 2515] (n)			
**• No**	1409 (98.1)	1069 (99.1)	1.00
**• Yes**	27 (1.9)	10 (0.9)	2.05 [0.89–4.72]
**Renal disease** [n = 2515] (n)			
**• No**	1417 (98.7)	1069 (99.1)	1.00
**• Yes**	19 (1.3)	10 (0.9)	1.43 [0.66–3.10]
**Lung diseases** [n = 2515] (n)			
**• No**	1414 (98.5)	1066 (98.8)	1.00
**• Yes**	22 (1.5)	13 (1.2)	1.28 [0.69–2.37]
**Epilepsy** [n = 2515] (n)			
**• No**	1426 (99.3)	1075 (99.6)	1.00
**• Yes**	10 (0.7)	4 (0.4)	1.88 [0.57–6.22]
**Systemic lupus erythematosus** [n = 2515] (n)			
**• No**	1423 (99.1)	1077 (99.8)	1.00
**• Yes**	13 (0.9)	2 (0.2)	**4.92 [1.21–19.98]**
**Thrombophilia or Thrombosis** [n = 2515] (n)			
**• No**	1429 (99.5)	1070 (99.2)	1.00
**• Yes**	7 (0.5)	9 (0.8)	0.58 [0.16–2.13]
**Oligohydramnios** [n = 2430] (p)			
**• No**	1034 (74.2)	987 (95.2)	1.00
**• Yes**	359 (25.8)	50 (4.8)	**6.85 [4.54–10.35]**
**Polyhydramnios** [n = 2430] (p)			
**• No**	1338 (96.1)	1020 (98.4)	1.00
**• Yes**	55 (3.9)	17 (1.6)	**2.47 [1.27–4.79]**
**Fetal malformation** [n = 2446] (q)			
**• No**	1277 (92.1)	1043 (98.4)	1.00
**• Yes**	109 (7.9)	17 (1.6)	**5.24 [2.57–10.68]**
**Fetal growth restriction** [n = 2446] (q)			
**• No**	1048 (75.6)	1033 (97.5)	1.00
**• Yes**	338 (24.4)	37 (2.5)	**12.34 [5.70–26.73]**
**Multiple pregnancy** [n = 2614]			
**• No**	1355 (92.3)	1136 (99.1)	1.00
**• Yes**	113 (7.7)	10 (0.9)	**9.47 [3.66–24.54]**
**Total**	**1468 (100)**	**1,146 (100)**	

OR*: Odds Ratio adjusted for the cluster effect design; **CI:** confidence interval; **PHU:** Primary Health Unit.

Values in bold mean they are statistically significant.

Missing information for: (a) 351; (b) 246; (c) 331; (d) 319; (e) 418; (f) 19; (g) 504; (h)1451; (i) 323; (j) 266; (k) 299; (l) 4; (m) 43; (n) 99; (o) 2; (p) 184; (q) 168.

Finally, results of multivariate analysis are in Table 6. Preeclampsia/eclampsia/HELLP (OR_adj_ 15.35; 95%CI 6.57–35.88) or multiple pregnancy (OR_adj_ 12.49; 4.56–32.05) were the highest risk factors for pi-PTB. Other hypertensive disorders (chronic hypertension and gestational hypertension), maternal morbidities (chronic diabetes, cardiac disease and systemic lupus erythematosus) and vaginal bleeding during pregnancy also showed higher risks for pi-PTB. The higher the maternal age or initial BMI, the higher the pi-PTB risk, whereas the higher the final BMI, the lower the risk for pi-PTB. Non-white skin colour was identified as factor associated with a lower pi-PTB risk.

**Table 6:** Variables independently associated with provider-initiated preterm birth in all women studied: multiple analyses by non-conditional logistic regression [n = 2059].

Variables	OR_adj_	95% CI	p-value
Chronic hypertension	7.47	[4.02–13.88]	<0.001
Preeclampsia/Eclampsia/HELLP	15.35	[6.57–35.88]	<0.001
Final BMI (kg/m^2^)	0.87	[0.83–0.92]	<0.001
Multiple pregnancy	12.49	[4.86–32.05]	<0.001
Chronic diabetes	5.24	[2.68–10.25]	<0.001
Initial BMI (kg/m^2^)	1.12	[1.06–1.19]	<0.001
Gestational hypertension	3.87	[1.98–7.57]	<0.001
Vaginal bleeding	1.64	[1.17–2.31]	0.006
Age (years)	1.02	[1.01–1.04]	0.017
Cardiac disease	2.76	[1.14–6.71]	0.027
Systemic lupus erythematosus	4.01	[1.08–14.85]	0.039
Skin colour/ethnicity (other: no white)	0.69	[0.49–0.98]	0.041

**OR**_**adj**_: Odds ratio adjusted for all predictors in this final model; **CI:** confidence interval of OR; **p:** p-value.

**Initial predictors entering the model:** age (years); skin colour/ethnicity (white: 0/ other: 1); marital status (with a partner: 0/ without a partner: 1); household (urban: 0/ rural: 1); paid work in pregnancy (yes: 1/ no: 0); children under 5 years (no: 0/ yes: 1); parity (until 2: 0/ ≥ 3: 1); prenatal care (yes: 0/ no: 1); weight gain at pregnancy (kg); initial BMI (kg/m^2^); final BMI (kg/m^2^); smoking (daily) (no: 0/ yes, ≥ 1 cigarettes: 1); smoking until (never or not in pregnancy: 0/ first to third trimester: 1); antenatal substance abuse (never: 0/ yes or before pregnancy: 1); periodontal infection (yes: 1/ no: 0); vaginal bleeding (yes: 1/ no: 0); anaemia (yes: 1/ no: 0); chronic hypertension (yes: 1/ no: 0); chronic diabetes (yes: 1/ no: 0); gestational diabetes (yes: 1/ no: 0); gestational hypertension (yes: 1/ no: 0); preeclampsia/eclampsia/HELLP (yes: 1/ no: 0); hypo/hyperthyroidism (yes: 1/ no: 0); HIV (yes: 1/ no: 0); cardiac disease (yes: 1/ no: 0); renal disease (yes: 1/ no: 0); lung diseases (yes: 1/ no: 0); epilepsy (yes: 1/ no: 0); systemic lupus erythematosus (yes: 1/ no: 0); thrombophilia or thrombosis (yes: 1/ no: 0); multiple pregnancy (yes: 1/ no: 0).

Variables included after the adjustment of the model due to the number of missing values (all non-significant): Schooling (<12:1/≥12:0); vulvovaginitis (bacterial vaginosis: 1/ no: 0); vulvovaginitis (candidiasis: 1/ no: 0); urinary tract infection (yes: 1/ no: 0); short cervix (yes: 1/ no: 0); cervical insufficiency (yes: 1/ no: 0); cerclage (yes: 1/ no: 0); uterine fibroid (yes: 1/ no: 0).

## Discussion

The EMIP study corroborates a recent upward trend in national and global preterm rates [2,4]. Previous studies have shown that pi-PTB is responsible for almost one-third of preterm births in Latin America. In higher-income populations with better access to health care, pi-PTB has increased, remaining the fastest growing determinant of preterm birth in recent decades [3,6,7,25]. Consistent recognition of pregnancy complications and maternal morbidities using screening protocols could partially justify the higher pi-PTB rates in middle and high-income settings.

Pi-PTB is not only the result of provider preference when faced with either pregnancy complications or prematurity consequences. It can also be a life-saving decision for both the mother and fetus. Pregnancy interruption seems more reasonable in the “near term” (34–36 weeks) group. Almost 60% of all pi-PTB currently occurs in this gestational age group. Nevertheless, this decision-making emblematic scenario in the late preterm group requires careful attention, since previous reports have described that more than 50% of late pi-PTB indications were not evidence-based [26]. In our data, less than 50% of pi-PTB due to fetal compromise had the support of Doppler ultrasound or cardiotocography. Only about one-quarter of pi-PTB due to maternal conditions were supported by maternal exams/tests, highlighting the importance of evidence-based decisions.

Obesity and advanced maternal age are factors related to a higher risk of preeclampsia, diabetes, chronic hypertension and other pregnancy complications or chronic diseases, and also resultant pi-PTB [27–31]. The higher the BMI before pregnancy, the higher the pi-PTB risk [30–33]. In contrast, underweight women before pregnancy and maternal age < 19 were related to a higher sPTB risk for the EMIP population [2], but not for pi-PTB. Advanced maternal age also increases the need for assisted fertility treatments, elevating the prevalence of multiple pregnancy, an important condition for both pi-PTB and sPTB [4]. Brazilian women conceive at increasingly older ages. In overweight or obese women, the scenario is unfavourable for a healthy pregnancy [34,35]. Preconception care package including nutritional, educational and family planning programs should be considered to prevent this situation [18].

Management of the clinical condition leading to pregnancy interruption was performed in more than 50% of cases. The most commonly treated or prevented maternal conditions were decompensated hypertension and diabetes [36,37]. However, the prevention of complications exclusively related to fetal conditions seems limited [38,39].

Caesarean section was significantly more frequent in pi-PTB than in term births, reaching 91.3% between 28 and 31 weeks. This potentially life-saving therapeutic mode of delivery increases maternal and neonatal morbidity and mortality [40]. Some women and/or fetuses are already at risk when a pi-PTB is indicated, therefore additional risk factors should be only considered when an evidence-based indication exists.

In the current study, more than half of pi-PTB occurred in conditions defined as ischemic placental disease (IPD), including preeclampsia, eclampsia, HELLP syndrome, fetal growth restriction, chronic hypertension, and placental abruption. IPD culminates in uteroplacental underperfusion, a phenomenon mediated by many biochemical angiogenic factors and autoimmune response regulators that are associated with its pathophysiology [41]. The hypertensive disorders of pregnancy (HDP) is one of the most important “Great Obstetrical Syndromes”, which is defined as being a condition with multiple aetiologies, having a long preclinical period, being adaptive in nature, resulting in fetal involvement and being a result of complex interaction between maternal and fetal genome and the environment [42]. HDP comprises four categories as chronic hypertension, gestational hypertension, preeclampsia/eclampsia and preeclampsia superimposed on chronic hypertension. They are related with adverse maternal and perinatal outcomes [43]. Additionally, preeclampsia, eclampsia and chronic hypertension are of great importance for pi-PTB occurrence. Two recent WHO studies, the Multicountry Survey and Global Survey, showed that those HDP are the main risk factors for pi-PTB [6,7]. In the current study, the majority of indications was related to hypertensive disorders, which highlights its importance in the context of preterm birth. Further studies of IPD, especially those related to hypertensive disorders of pregnancy, could help prevent this condition [44,45].

It is also important to compare factors associated with sPTB to those related to pi-PTB in order to identify interrelationships between such complex outcomes. The population at higher risk for each condition, neonatal outcomes and risk factors may vary depending on the main determinant (spontaneous onset, pPROM or pi-PTB) [4,6,7,25]. Previous analyses of EMIP showed that maternal age and years of schooling were the main factors associated with sPTB [2]. It was demonstrated that older white women, with higher initial BMI and schooling had more pi-PTB. This is not an indubitable phenotypic risk. A high-risk group could be at increased risk for pi-PTB, and receive more specialized care [6]. These women have more access to prenatal care, adhering strictly to medical appointments. Owing to higher education, they have a better self-perception of risk, undergoing more fertility treatments due to advanced age, resulting in a higher proportion of multiple births.

Some possible limitations of this study do exist. First, the evaluation of antenatal corticosteroid use according to WHO gestational age subcategories could be misinterpreted. The percentage of corticosteroid use was probably underestimated in women with pi-PTB below 28 weeks because there is no evidence-based indication below 24 weeks [46]. In fact, none of these women received ACS (n = 11, data not shown). Additionally, the moderate to late preterm subcategory (32–36 weeks) includes gestational age when ACS use is not recommended (34–36), underestimating its use. However, ACS was used in almost three-quarters of the 28–31 week group. Although this is not yet an ideal rate for a pi-PTB, it was significantly higher than ACS provided to one-third of preterm births in Brazil, as reported by the WHO Multicountry Survey [47]. It is worth mentioning that some pi-PTBs had urgent indication, with insufficient time for a complete ACS regimen. Second, although the study was conducted in 20 referral facilities throughout Brazil, definitely this is not a population study. The higher prevalence of high-risk pregnant women in participating centres weakened sample representativeness for the entire Brazilian population. Finally, weight gain during pregnancy would be better assessed if the weight gain pattern had been used separately by different BMI categories, since suitability of weight gain depends on the initial maternal BMI [48]. This point is planned to be the focus for a further in depth secondary analysis, as is also the case for fetal malformations, multiple pregnancies and some other very specific conditions.

Neonatal outcomes in pi-PTB are dependent on some antenatal factors, including gestational age, multiple pregnancies, ACS use, maternal complications (preeclampsia, HELLP syndrome, chronic hypertension, diabetes, infection, etc.) and fetal complications (malformation, FGR or fetal distress). In the WHO Multicountry Survey, the risk of early neonatal death in sPTB infants was shown to be lower than in pi-PTB infants [49]. Maternal and fetal compromise before delivery may determine worse outcomes. The higher prevalence (6-fold) of late preterm neonatal death compared to term neonates indicate that an alternative approach for decision-making strategies and high-quality neonatal support for this group should be considered [26].

The main strengths of EMIP study were the high number of cases, and validation and crosschecking rules of data collection for more than 300 variables, allowing analysis of the Brazilian preterm background with high-quality reliable data. To the best of our knowledge, there is no other official data on determinants of preterm birth in Brazil with such a comprehensive approach. Therefore, the EMIP database is extremely important to assess conditions related to pi-PTB, at least for the Brazilian population. Recognition of conditions related to pi-PTB, evaluation of perinatal outcomes, reassessment of correlated clinical practices and management protocols are the key to making progress in primary, secondary and tertiary preventions of preterm birth [15,17,18].

### What is already known on this subject

Provider-initiated preterm birth is gaining more importance in recent decades, since major efforts to prevent preterm births have achieved limited results. The proportion of pi-PTB has increased with decreasing neonatal mortality and increasing Human Development Index. Some conditions, including advanced maternal age, preeclampsia, previous caesarean section, placenta previa and hypertension were identified as risk factors for pi-PTB. There is no previous comprehensive study specifically exploring this preterm birth subtype and its associated factors. In-depth knowledge of underlying conditions associated with pi-PTB is essential to improve preventive strategies for pi-PTB and consequently preterm birth, that no longer occur only in high-income, but also in low and middle-income settings.

### What this study adds

Identifying factors associated with pi-PTB contributes to strategies for preterm birth prevention, including preconception care package, high-risk pregnancy screening, and human and material resources required for obstetric and neonatal care facilities. The proportion of pi-PTB is also already high among all preterm births in a middle-income country, as probably occurs in other similar settings. The need for future larger studies is highlighted to build a real profile of these conditions and understand how to implement primary and secondary pi-PTB prevention.

## Supporting Information

S1 Table Dataset for provider-initiated preterm birth. (XLSX)

S2 Table STROBE Checklist. (DOC)
